# Sitafloxacin-Containing Regimen for the Treatment of Refractory *Mycobacterium avium* Complex Lung Disease

**DOI:** 10.1093/ofid/ofz108

**Published:** 2019-03-07

**Authors:** Takanori Asakura, Shoji Suzuki, Hanako Fukano, Satoshi Okamori, Tatsuya Kusumoto, Yoshifumi Uwamino, Takunori Ogawa, Matsuo So, Shunsuke Uno, Ho Namkoong, Mitsunori Yoshida, Hirofumi Kamata, Makoto Ishii, Tomoyasu Nishimura, Yoshihiko Hoshino, Naoki Hasegawa

**Affiliations:** 1 Department of Mycobacteriology, Leprosy Research Center, National Institute of Infectious Diseases; 2 Division of Pulmonary Medicine, Department of Medicine, Keio University School of Medicine; 3 Japan Society for the Promotion of Science, Tokyo; 4 Department of Laboratory Medicine, Keio University School of Medicine; 5 Center for Infectious Diseases and Infection Control, Keio University School of Medicine; 6 Keio University Health Center, Tokyo

**Keywords:** clarithromycin (CLR) resistance, difficult to treat, fluoroquinolone, *Mycobacterium avium* complex (MAC), nontuberculous mycobacteria (NTM)

## Abstract

**Background:**

Sitafloxacin (STFX) exhibits potent activity against *Mycobacterium avium* complex (MAC) in both in vitro and in vivo experiments. However, limited data are available for the clinical efficacy and adverse effects of STFX and the susceptibility of refractory MAC lung disease (MAC-LD) to the drug. Therefore, this study was aimed at evaluating the clinical efficacy and safety of an STFX-containing regimen for the treatment of refractory MAC-LD.

**Methods:**

We retrospectively evaluated treatment outcomes of 31 patients with refractory MAC-LD, who received an STFX-containing regimen for ≥4 weeks between January 2010 and July 2017. Refractory MAC-LD was defined as persistent positive sputum cultures for >6 months of macrolide-based standard therapy.

**Results:**

Clarithromycin resistance (minimum inhibitory concentration [MIC] ≥32 μg/mL) was identified in 15 patients (48%). Twelve months after receiving the STFX-containing regimen, 26% and 19% of patients showed symptomatic and radiological responses, respectively. Although STFX-associated adverse effects were noted in 9 patients, their severity was grade 1 (National Cancer Institute Common Terminology Criteria); only 1 patient discontinued STFX because of suspected gastrointestinal disturbance. Negative sputum culture conversion was achieved in 7 patients (23%). Both univariate and multivariate logistic regression analyses revealed that surgery, low STFX MIC (≤1 μg/mL), and macrolide resistance were significant predictors of negative sputum culture conversion.

**Conclusions:**

Our results demonstrate that STFX may be effective in one-fourth of patients with refractory MAC-LD. Prospective larger studies that include the analyses of MAC are needed to determine the clinical efficacy of STFX against refractory MAC-LD.

## Teaser

Our results demonstrate that sitafloxacin (STFX) may be effective in one-fourth of patients with refractory *Mycobacterium avium* complex lung disease. Surgery, low minimum inhibitory concentration of STFX, and clarithromycin resistance were predictors of negative sputum culture conversion.

The incidence of pulmonary disease caused by nontuberculous mycobacteria has been increasing worldwide [1, 2]. *Mycobacterium avium* complex (MAC) is the most common pathogen causing nontuberculous mycobacteria lung disease in Japan [3]. MAC lung disease (MAC-LD) mostly causes chronic progressive disease, resulting in impaired quality of life and survival [4, 5]. Although multiple antimicrobial therapies, including macrolide-containing regimens, have been developed and recommended in the last decade [6], their efficacy is often limited, especially in patients with advanced MAC-LD [7–9]. The treatment success rate, reflected by the sputum culture conversion, is approximately 50%–60% [7–9]. In addition to treatment failure, relapse or acquired clarithromycin (CLR) resistance makes MAC-LD treatment more difficult.

Quinolone antimicrobials inhibit the maintenance of chromosomal topology by targeting both DNA gyrase and topoisomerase IV [10]. Previous studies revealed that some fluoroquinolones were clinically used for the initial treatment or the treatment of relapsed/refractory MAC-LD [11–16]. Sitafloxacin (STFX), a C-8 chloroquinolone, is a newly developed oral quinoline, exhibiting potent activity against MAC both in vitro and in vivo [17, 18]. Recently, a case series (including 4 patients with relapsed and 14 with refractory MAC-LD) revealed the potential use of STFX for relapsed or refractory MAC-LD [16]. However, there are limited data regarding the clinical efficacy and adverse effects of STFX, as well as the susceptibility of MAC-LD to STFX. Therefore, in the current study, we aimed to evaluate the clinical efficacy and safety of STFX-containing regimens for the treatment of MAC-LD.

## METHODS

### Study Population and Antibiotic Treatment

We identified all patients who were administered STFX from prescription records between January 2010 and July 2017 at Keio University Hospital (a 1044-bed referral hospital in Tokyo, Japan). Of the 183 patients who received STFX, we identified 31 with refractory MAC-LD who received an STFX-containing regimen for ≥4 weeks. All patients satisfied the 2007 American Thoracic Society/Infectious Diseases Society of America diagnostic criteria [6] and tested negative for anti–human immunodeficiency virus antibody. Refractory MAC-LD was defined as persistent positive sputum culture results for >6 months of standard therapy, which was considered to be macrolide-based multidrug treatment. All patients were treated with STFX for the first time, usually prescribed at a dosage of 200 mg/d; some patients (eg, elderly patients and those with low body weight) were prescribed a lower dosage, as instructed by the attending physicians. The ethics review board of Keio University Hospital approved the retrospective study protocol (no. 20080131). Informed consent was waived because of the retrospective nature of the assessment.

### Clinical Evaluation, Including Radiological and Microbiological Findings

The evaluated baseline clinical characteristics included age, sex, disease duration, body mass index, smoking status, underlying pulmonary disease, radiological findings, results of pulmonary function tests, and sputum smear and culture results for MAC. All patients underwent chest radiography or high-resolution computed tomography (HRCT) at the time of initial STFX treatment and during follow-up. Radiographic patterns were classified into 4 forms (nodular/bronchiectatic [NB], fibrocavitary [FC], NB and FC, and unclassified), according to findings in previous studies [19].

MAC isolates were identified as described elsewhere [20]. A test for susceptibility to CLR, rifampin (RIF), ethambutol (EMB), amikacin (AMK), levofloxacin (LVX), moxifloxacin (MXF), and STFX was performed at the National Institute of Infectious Diseases, using the broth microdilution method as described by the Clinical and Laboratory Standards Institute [21]. The range of concentrations of the tested drugs was 0.0625–64 μg/mL, except for AMK (range, 0.25–64 μg/mL). A minimum inhibitory concentration (MIC) ≥32 μg/mL for CLR was defined as resistance.

### Evaluation of Treatment Response and Adverse Effects

We evaluated treatment response 12 months after starting the STFX treatment, and patients with <12 months of treatment were evaluated based on their data at the last follow-up. Symptomatic and radiological responses were evaluated using medical records, which included initial and follow-up chest radiographic and HRCT findings and their classification by 2 pulmonologists (TA and SS) as improved, unchanged, or worsened. Any discrepancy was resolved by an additional investigator and by consensus review [22]. Negative sputum culture conversion was defined as 3 consecutive negative sputum cultures [23, 24], and if the patients did not expectorate sputum, the status was recorded as negative [25]. Microbiological cure and recurrence (in patients with negative sputum culture conversion) were defined as no positive cultures after culture conversion until the end of treatment and reemergence of multiple positive cultures after cessation of treatment, respectively [24]. The time to conversion was defined as the date of the first negative culture. All adverse events were graded according to National Cancer Institute Common Terminology Criteria for adverse events (version 4.0).

### Statistical Analysis

The data for continuous variables are expressed as median (interquartile range [IQR]), and those for categorical variables as number (percentage). The Fisher exact test was used for comparisons between 2 groups. To determine the factor associated with successful sputum culture conversion, a univariate logistic regression analysis was performed. A multivariate logistic regression analysis was also performed using the significant variables determined by the univariate analysis. Differences were considered statistically significant at *P* < .05. Analyses were performed using JMP software (version 14.0; SAS Institute Japan).

## RESULTS

### Patient Characteristics

Table 1 shows the baseline characteristics of 31 patients at the time of initiation of STFX treatment. The median (IQR) patient age was 68 (63–77) years, and the majority of patients (81%) were female. Only 4 patients (13%) had underlying pulmonary disease. Chest HRCT showed that 17 patients (55%) had the NB form of MAC-LD, 13 (42%) had the NB and FC form, and 1 (3%) had an unclassified form. Cavitary lesions were found in 11 patients (35%). Regarding pulmonary function, the median (IQR) percentage of predicted forced expiratory volume in 1 second (FEV_1_) was 74 (55–93)%; the median percentage of predicted forced vital capacity (FVC), 84 (66–96)%; the median FEV_1_/FVC, 75 (68–80)%. Acid-fast bacillus smears of sputum were positive in 22 patients (65%).

**Table 1.** Baseline Characteristics at Initiation of Sitafloxacin Therapy

Clinical Characteristics	Patients, No. (%)^a^ (n = 31)
Age, median, y	68 (63–77)
Female sex	25 (81)
Disease duration, median, y	7.5 (4.5–13.3)
Body mass index, median, kg/m^2^	18.1 (16.7–19.9)
Smoking status	
Never/former	30 (97)/1 (3)
Underlying pulmonary disease^b^	
History of pulmonary tuberculosis	1 (3)
Asthma	1 (3)
Chronic obstructive pulmonary disease	1 (3)
Chronic pulmonary aspergillosis	1 (3)
*Mycobacterium avium*/*intracellulare*	28 (90)/3 (10)
Concomitant *Pseudomonas aeruginosa*	3 (10)
Radiological findings	
NB/FC/NB+FC/unclassified	17 (55)/0 (0)/13 (42)/1 (3)
Presence of cavity	11 (35)
No. of involved lobes, median	6 (5–6)
Pulmonary function test result, median	
FEV_1_, % of predicted	74 (66–93)
FVC, % of predicted	84 (66–96)
FEV_1_/FVC, %	75 (68–80)
AFB smear–positive sputum	20 (65)

Abbreviations: AFB, acid-fast bacillus; FC, fibrocavitary; FEV_1_, forced expiratory volume in 1 second; FVC, forced vital capacity; IQR, interquartile range; NB, nodular/bronchiectatic.

^a^Data represent no. (%) of patients unless identified as median (IQR).

^b^No patient had interstitial lung disease or lung cancer.

Table 2 shows the results of the drug susceptibility test at the time of initiation of STFX treatment. Macrolide resistance was identified in 15 patients (48%). Distribution of the STFX MIC tended to be lower than that of LVX and MXF.

**Table 2.** In Vitro Susceptibility of *Mycobacterium avium* Complex Isolates (n = 31)

		No. of Strains by MIC (μg/mL)												
Drug	Drug Concentration, μg/mL	<0.0625	0.0625	0.125	0.25	0.5	1	2	4	8	16	32	64	>64
CLR	0.0625–64	1	0	0	0	3	4	4	2	2	0	2	0	13
EMB	0.0625–64	0	0	0	0	0	0	1	5	2	4	7	5	7
RIF	0.0625–64	1	1	1	1	1	0	6	5	3	2	5	4	1
AMK	0.25–64	0	0	0	0	0	1	3	0	13	8	3	1	2
LVX	0.0625–64	0	0	1	0	3	1	8	4	2	8	3	0	1
MXF	0.0625–64	0	0	4	0	6	10	7	3	0	0	0	1	0
STFX	0.0625–64	4	0	3	4	8	4	5	3	0	0	0	0	0

Abbreviations: AMK, amikacin; CLR, clarithromycin; EMB, ethambutol; LVX, levofloxacin; MIC, minimum inhibitory concentration; MXF, moxifloxacin; RIF, rifampin; STFX, sitafloxacin.

### Antibiotic Treatment Regimens

Table 3 shows the antibiotics received before and after STFX treatment. All patients who received STFX-containing regimens had persistent positive cultures after previous therapy for a median (IQR) of 60 (34–104) months. Fourteen patients (45%) had received 1 regimen and 17 (55%) had received ≥2. Streptomycin or kanamycin was injected intramuscularly before STFX treatment in 5 patients (16%), and AMK was injected intravenously injection or administered as inhalation therapy in 11 (35%). After initiation of STFX treatment, CLR was continued in 19 patients (61%); in the other 12 (39%), CLR was discontinued owing to the development of CLR resistance. Most companion drugs, such as EMB (22 patients [71%]) and RIF (25 patients [81%]), were continued. STFX was prescribed at a dosage of 200 mg/d in 28 patients (90%) and 100 mg/d in 3 (10%). During the 12 months after STFX initiation, AMK was administered as an intravenous injection in 7 patients (23%) for a median (IQR) of 4.1 (1–6.1) months and as inhalation therapy in 7 patients (23%) for 5.8 (5.2–6.8) months. Two patients received AMK intravenous injection after the AMK inhalation therapy. 

**Table 3.** Treatment Regimen for *Mycobacterium avium* Complex Lung Disease

Treatment	Patients, No. (%)^a^ (n = 31)
Before sitafloxacin treatment	
Clarithromycin	31 (100)
Ethambutol	28 (90)
Rifampin	27 (87)
Rifabutin	1 (3)
Ciprofloxacin	2 (6)
Levofloxacin	6 (19)
Garenoxacin	2 (6)
Moxifloxacin	3 (10)
Streptomycin or kanamycin injection	5 (16)
Amikacin injection	7 (23)
Amikacin inhalation	4 (13)
Surgery	2 (6)
Total treatment duration, median, mo	60 (34–104)
After sitafloxacin treatment	
Clarithromycin	19 (61)
Ethambutol	22 (71)
Rifampin	25 (81)
Amikacin injection	7 (23)
Amikacin inhalation	7 (23)
Sitafloxacin	31 (100)
200 mg/d	28 (90)
100 mg/d	3 (10)
Surgery	3 (10)
Duration of sitafloxacin treatment, median, mo	12 (12–12)

Abbreviation: IQR, interquartile range.

^a^Data represent no. (%) of patients unless identified as median (IQR).

Regarding the treatment for patients with or without CLR resistance, CLR was used more in CLR-susceptible patients (CLR susceptibility and resistance, 14 patients [88%] and 5 patients [33%], respectively; *P* = .003). However, the number of patients who received AMK inhalation or injection (CLR susceptibility and resistance, 7 patients [43%] and 5 patients [33%]; *P* = .72), or underwent surgery (2 patients [13%] and 1 patient [6%], respectively; *P* = .60) did not differ between the 2 groups. Surgery was performed in 3 patients, 2 weeks, 3 months, and 9 months after the addition of STFX. Only 4 patients received STFX treatment for <12 months. The proportion of AMK use or surgery did not differ significantly between patients with and those without CLR resistance (AMK use, *P* = .72; surgery, *P* = .60).

### Treatment Outcomes and Adverse Effects After STFX Treatment for 12 Months

Table 4 shows the treatment responses after the initiation of STFX treatment. There was symptomatic improvement in 8 patients (26%) and radiological improvement in 6 (19%). Nine patients (29%) showed either symptomatic or radiological improvement (both, 5 patients; symptomatic improvement only, 3; radiological improvement only, 1). Twelve patients (39%) had ≥1 negative culture, and negative sputum culture conversion was noted in 7 (23%). Of these 7 patients with negative sputum culture conversion, microbiological cure was achieved in 5 (71%), and 2 (29%) had recurrence. Regarding emergent CLR resistance, in the follow-up of 16 patients with CLR-susceptible MAC, CLR susceptibility was evaluated in 15 of them, and CLR resistance developed in 4 (27%), who did not achieve negative sputum culture conversion. 

**Table 4.** Treatment Response 12 Months After Initiation of Sitafloxacin Treatment

Treatment Response	Patients, No.^a^ (%) (n = 31)
Symptomatic responses improved/unchanged/worsened	8 (26)/13 (42)/10 (32)
Radiological responses improved/unchanged/worsened	6 (19)/9 (29)/16 (52)
≥1 Negative culture	12 (39)
Sputum culture conversion	7 (23)
Lost to follow-up	2 (6)
Death	2 (6)
Duration of follow-up, median, mo	12 (12–12)

Abbreviation: IQR, interquartile range.

^a^Data represent no. (%) of patients unless identified as median (IQR).

Two patients (6%) were lost to follow-up and died during the 12 months after STFX administration. Adverse effects associated with STFX occurred in 9, including gastrointestinal disturbance in 5 patients (16%), rash in 2 (6%), liver dysfunction in 1 (3%), and hemoptysis in 1(3%). The severity of the adverse effects was grade 1. Only 1 patient discontinued STFX, 1 month after treatment initiation, owing to gastrointestinal disturbance; in the other 4 patients, gastrointestinal disturbance improved spontaneously. In the 2 patients with rash, 1 was treated successfully with an antihistamine and the other’s rash improved spontaneously. In the patient with liver dysfunction, STFX was temporally discontinued but then resumed safely because RIF was determined to be the cause of the hepatic dysfunction. Among the 3 patients who received STFX at 100 mg/d, a rash developed in 1.

### Predictors of Sputum Culture Conversion

The Supplementary Material shows the in vitro MIC of STFX in MAC isolates and the rate of sputum culture conversion. Patients in whom the MIC was >1 μg/mL did not show sputum culture conversion. The proportion of isolates with STFX MIC ≤1 μg/mL tended to be higher in patients with CLR resistance, but this difference was not statistically significant (*P* = .11). Table 5 shows the results of the univariate and multivariate analyses for the predictors of sputum culture conversion in patients with MAC-LD. In the univariate analysis, macrolide resistance (odds ratio [OR], 10.0; 95% confidence interval [CI], 1.03−97.0; *P* = .02), STFX MIC ≤1 μg/mL (OR not available; *P* = .03), and surgery (OR not available; *P* = .001) were predictors of negative sputum culture conversion. If we exclude the 3 patients who underwent surgery, macrolide resistance (OR not available; *P* = .008) was a predictor of negative sputum culture conversion, but STFX MIC ≤1 μg/mL was not (OR not available; *P* = .09). 

**Table 5.** Predictors of Negative Sputum Culture Conversion in Patients With *Mycobacterium avium* complex Lung Disease Treated With a Sitafloxacin-Containing Antibiotic Regimen^a^

Predictor	Sputum Culture Conversion, No. (%) of Patients		Univariate Analysis		Multivariate Analysis	
Characteristics	Yes (n = 7)	No (n = 24)	OR (95% CI)	*P* Value	aOR (95% CI)	*P* Value
Age						
≥65 y	5 (71)	14 (58)	Reference	.53	…	…
<65 y	2 (29)	10 (42)	0.56 (.09–3.49)		…	
Sex						
Male	2 (29)	4 (17)	Reference	.50	…	…
Female	5 (71)	20 (83)	0.50 (.07–3.55)		…	
BMI						
≤18.5 kg/m^2^	4 (57)	15 (62)	Reference	.80	…	…
>18.5 kg/m^2^	3 (43)	9 (38)	1.25 (.23–6.91)		…	
Radiographic patterns						
Other	2 (29)	12 (50)	Reference	.31	…	
NB form	5 (71)	12 (50)	2.50 (.40–15.5)		…	
Cavitary lesion						
Yes	3 (43)	17 (71)	Reference	.18	…	…
No	4 (57)	7 (29)	3.23 (.57–18.4)		…	
Sputum smear for AFB						
Positive	5 (71)	15 (62.5)	Reference	.67	…	…
Negative	2 (29)	9 (37.5)	0.67 (.11–4.18)		…	
AMK use (injection or inhalation)						
No	3 (43)	16 (67)	Reference	.26	…	…
Yes	4 (57)	8 (33)	2.67 (.48–14.9)		…	
EMB or RIF MIC						
≥8 μg/mL	3 (43)	9 (37.5)	Reference	.80	…	…
<8 μg/mL	4 (57)	15 (62.5)	0.80 (.14–4.42)		…	
Treatment duration						
≥12 mo	6 (86)	21 (87.5)	Reference	.90	…	…
<12 mo	1 (14)	3 (12.5)	1.17 (.10–13.4)		…	
STFX dosage 200 mg/d						
No	0 (0)	3 (12.5)	Reference	.20	…	…
Yes	7 (100)	21 (87.5)	NA		…	
Macrolide resistance						
No	1 (14)	15 (62)	Reference	.02	Reference	.001
Yes	6 (86)	9 (38)	10.0 (1.03–97.0)		NA	
STFX MIC						
>1 μg/mL	0 (0)	8 (33)	Reference	.03	Reference	.01
≤1 μg/mL	7 (100)	16 (67)	NA		NA	
Surgery						
No	4 (57)	24 (100)	Reference	.001	Reference	.003
Yes	3 (43)	0 (0)	NA		NA	

Abbreviations: AFB, acid-fast bacilli; AMK, amikacin; aOR, adjusted odds ratio; BMI, body mass index; CI, confidence interval; EMB, ethambutol; MIC, minimum inhibitory concentration; NA, not available; NB, nodular/bronchiectatic; OR, odds ratio; RIF, rifampin; STFX, sitafloxacin.

^a^No stable estimate was obtained because in all patients in whom the STFX MIC was >1 μg/mL, sputum culture conversion did not occur; sputum culture conversion occurred in all patients who underwent surgery; and sputum culture conversion occurred in patients who underwent surgery, whose STFX MIC was ≤1 μg/mL, or who had macrolide resistance.

Multivariate analysis revealed that macrolide resistance (adjusted OR not available; *P* = .001), STFX MIC ≤1 μg/mL (adjusted OR not available; *P* = .01), and surgery (OR not available; *P* = .003) were also predictors of negative sputum culture conversion. In addition, stratified analysis (Supplementary Material) with macrolide resistance revealed that STFX MIC ≤1 μg/mL (OR not available; *P* = .003) and surgery (OR not available; *P* = .04) were predictors of negative sputum culture conversion. Moreover, stratified analysis without macrolide resistance revealed that emergence of macrolide resistance was not a significant predictor of negative sputum culture conversion.

## DISCUSSION

This study evaluates the clinical efficacy and safety of an STFX-containing regimen for refractory MAC-LD by means of various approaches, including in vitro drug susceptibility tests. In one-fourth of the patients, negative sputum culture conversion was achieved without severe adverse effects. Moreover, in this cohort of individuals with existing refractory MAC-LD treated with STFX, in vitro susceptibility results for STFX (MIC ≤1 μg/mL) or CLR (MIC ≥32 μg/mL as resistant) were associated with sputum culture conversion in a multivariate analysis.

Fluoroquinolone-containing regimens have been used as alternative options for the treatment of MAC-LD in various situations. Two studies, which included patients who received ciprofloxacin, ofloxacin, or LVX in combination with a macrolide, revealed rates of sputum culture conversion that were 68% and 84%, despite the previous treatment status being unknown [11, 13]. Another study revealed that a ciprofloxacin-containing regimen had no advantage over a macrolide-based therapy [12]. Regimens containing gatifloxacin (which is now being withdrawn from the market owing to adverse effects) showed high efficacy and relative safety in treatment-naive MAC-LD, similar to results of a CLR-based regimen. However, the sample size in that study was small, and the long-term risk for macrolide resistance was unknown [14].

Regarding refractory/relapsed MAC-LD, in a previous study with STFX-containing regimens, negative sputum culture conversion was achieved in 8 of 18 patients (44%), indicating better results than in our current study despite the lower dose of STFX used (50–100 mg) [16]. The rates of macrolide resistance and treatment duration before STFX administration were not reported in the earlier study. The resistance in our patients with MAC-LD might be more severe, with the disease being refractory in nature, because the previous study showed that 28% of patients had a treatment history of ≥2 regimens, lower than that the proportion in our study (55%). Another study using MXF-containing regimens for refractory MAC-LD demonstrated negative sputum culture conversion in 12 of 41 patients (29%), a slightly higher rate than that in our study [15]. The previous study had a higher proportion of patients with *Mycobacterium intracellulare* infection and the FC form of MAC-LD than did ours, suggesting a lower treatment response in the study of nonrefractory MAC-LD [26]. However, the much greater treatment duration before fluoroquinolone-containing regimens in our study could also affect the lower rate of negative sputum culture conversion.

Interestingly, STFX treatment was effective in patients whose infection was susceptible to STFX (MIC ≤1 μg/mL). In addition, the MIC for STFX may be responsible for the effectiveness in some patients with macrolide resistance (Supplementary Material). In the management of MAC-LD, the clinical utility of drug susceptibility tests has not been established except for CLR [27]. Recently, an MIC ≥8 μg/mL for both EMB and RIF reflected an unfavorable response to standard treatment for MAC-LD [28]; however, it was not associated with negative sputum conversion in our patients with refractory MAC-LD. With regard to fluoroquinolone, MXF susceptibility break points, defined only by the Clinical and Laboratory Standards Institute, were not predictors of treatment response for refractory MAC-LD [15]. Although STFX break points have not been established, our results suggest an association between in vitro susceptibility and treatment outcome.

In our study, the STFX-containing regimen showed a higher rate of negative sputum culture conversion in refractory MAC-LD with CLR resistance (MIC ≥32 μg/mL) than in patients infected with CLR-susceptible strains (6 of 15 patients [40%] vs 1 of 16 [6%], respectively). Patients with macrolide-resistant (MR) MAC-LD showed poor treatment outcomes and increased mortality rates [29–31], especially those whose cultures remained positive after treatment [29]. Treatment for MR MAC-LD has not been established, and only parenteral aminoglycoside and surgery have been associated with good prognosis, although there are a limited number of surgical candidates [30]. 

Regarding fluoroquinolones, previous studies demonstrated that the addition of fluoroquinolones did not improve outcomes in MR MAC-LD [15, 30–32]. With regard to the types of fluoroquinolones, in Korean studies, the addition of MXF was rarely effective for MR MAC-LD [15, 31]. Another study including patients with MR MAC-LD who received LVX (16 patients), MXF (2 patients), or STFX (2 patients) showed that sputum culture conversion was achieved in 6 of 20 patients (30%) [32]. This finding was more similar to our result (6 of 15 patients [40%]), despite the lower in vitro and in vivo activities of LVX compared with of MXF and STFX [18]. Although comparisons are difficult owing to various differences, including sex, species, disease form, disease duration, and treatment regimens before and after the use of fluoroquinolone, our results indicate that STFX may be a treatment option for MR MAC-LD.

The present study has some limitations. First, it is a single-center retrospective analysis including a limited number of cases, too small to detect clinically significant predictors for negative sputum culture conversion. Second, there could be a selection bias because STFX-containing regimens were administered depending on the discretion of the attending physician. Third, we could not evaluate the efficacy of STFX alone because the combined use with AMK or surgical resection could have influenced the treatment outcomes. However, STFX may be beneficial for patients with refractory MAC-LD with macrolide resistance or low STFX MIC.

In conclusion, STFX administration may be effective in one-fourth of patients with refractory MAC-LD, especially those in whom the STFX MIC is low (≤1 μg/mL) or those who show CLR resistance. Larger prospective studies, including analyses of MAC, are needed to investigate the clinical efficacy of STFX against refractory MAC-LD.

## Supplementary Data

Supplementary materials are available at *Open Forum Infectious Diseases* online. Consisting of data provided by the authors to benefit the reader, the posted materials are not copyedited and are the sole responsibility of the authors, so questions or comments should be addressed to the corresponding author.

Supplementary Material

## Notes

***Potential conflicts of interest.*** All authors: No reported conflicts of interest. All authors have submitted the ICMJE Form for Disclosure of Potential Conflicts of Interest. The authors declare no conflicts of interest associated with this manuscript. All authors have submitted the ICMJE Form for Disclosure of Potential Conflicts of Interest. Conflicts that the editors consider relevant to the content of the manuscript have been disclosed.
