# Supplementary material for: Sitafloxacin-Containing Regimen for the Treatment of Refractory Mycobacterium avium Complex Lung Disease
Source: Open Forum Infect Dis. 2019 Mar 7;6(4):ofz108. doi: 10.1093/ofid/ofz108 (PMC6519390; doi:10.1093/ofid/ofz108)
Supplement: Supplementary Material [file ofz108_suppl_supplementary_material.doc]

**Supplementary Material**

**Sitafloxacin-containing Regimen for the Treatment of Refractory *Mycobacterium avium* complex Lung Disease**

Takanori Asakura, Shoji Suzuki, Hanako Fukano, Satoshi Okamori, Tatsuya Kusumoto, Yoshifumi Uwamino, Takunori Ogawa, Matsuo So, Shunsuke Uno, Ho Namkoong, Mitsunori Yoshida, Hirofumi Kamata, Makoto Ishii, Tomoyasu Nishimura, Yoshihiko Hoshino, Naoki Hasegawa

| **Supplementary Table 1.** *In vitro* MIC of sitafloxacin for *Mycobacterium avium* complex isolates and rate of sputum culture conversion (*n* = 31) | | | | | | | | |
| --- | --- | --- | --- | --- | --- | --- | --- | --- |
|  | MIC^a^ of sitafloxacin (μg/ml) | | | | | | | |
|  | <0.0625 | 0.0625 | 0.125 | 0.25 | 0.5 | 1 | 2 | 4 |
| No. of isolates (%) | 4 (13) |  | 3 (10) | 4 (13) | 8 (26) | 4 (13) | 5 (16) | 3 (10) |
| Percentage of sputum culture conversion | 25 |  | 0 | 25 | 50 | 25 | 0 | 0 |

^a^ MIC, minimum inhibitory concentration

| **Supplementary Table 2.** Predictors of negative sputum culture conversion in MAC-LD patients with/without clarithromycin resistance who were treated with a STFX-containing antibiotic regimen | | | | | | | | | | |
| --- | --- | --- | --- | --- | --- | --- | --- | --- | --- | --- |
|  | Macrolide resistance (-) (*n* = 16) | | | | | Macrolide resistance (+) (*n* = 15) | | | | |
|  | Sputum culture conversion | |  | Univariate analysis^a^ |  | Sputum culture conversion | |  | Univariate  analysis^a^ | |
| Characteristics | Yes (*n* = 1) | No (*n* = 15) |  | *P* value |  | Yes (*n* = 6) | No (*n* = 9) |  | OR (95% CI) | *P* value |
| Age ≥ 65 years | 1 (100) | 8 (53) |  |  |  | 4 (67) | 6 (67) |  | Ref |  |
| < 65 years | 0 (0) | 7 (47) |  | 0.273 |  | 2 (33) | 3 (33) |  | 1.00 (0.11–8.95) | 1.000 |
| Sex male | 0 (0) | 3 (20) |  |  |  | 2 (33) | 1 (11) |  | Ref |  |
| female | 1 (100) | 12 (80) |  | 0.512 |  | 4 (67) | 8 (89) |  | 0.25 (0.02–3.66) | 0.295 |
| BMI ≤ 18.5 kg/m^2^ | 1 (100) | 9 (60) |  |  |  | 3 (50) | 6 (67) |  | Ref |  |
| > 18.5 kg/m^2^ | 0 (0) | 6 (40) |  | 0.301 |  | 3 (50) | 3 (33) |  | 2.00 (0.24–16.6) | 0.519 |
| Radiographic patterns, Others | 0 (0) | 7 (47) |  |  |  | 2 (33) | 5 (56) |  | Ref |  |
| NB form | 1 (100) | 8 (53) |  | 0.273 |  | 4 (67) | 4 (44) |  | 2.50 (0.29–21.4) | 0.395 |
| Cavitary lesion (+) | 0 (0) | 10 (67) |  |  |  | 3 (50) | 7 (78) |  | Ref |  |
| (-) | 1 (100) | 5 (33) |  | 0.150 |  | 3 (50) | 2 (22) |  | 3.50 (0.37–33.0) | 0.265 |
| Sputum smear for AFB (+) | 1 (100) | 10 (67) |  |  |  | 4 (67) | 5 (56) |  | Ref |  |
| (-) | 0 (0) | 5 (33) |  | 0.377 |  | 2 (33) | 4 (44) |  | 0.63 (0.07–5.35) | 0.667 |
| AMK use (injection or inhalation) (-) | 0 (0) | 9 (60) |  |  |  | 3 (50) | 7 (78) |  | Ref |  |
| (+) | 1 (100) | 6 (40) |  | 0.187 |  | 3 (50) | 2 (22) |  | 3.50 (0.37–33.0) | 0.265 |
| EMB or RIF MIC ≥ 8 μg/ml | 0 (0) | 5 (33) |  |  |  | 3 (50) | 4 (44) |  | Ref |  |
| < 8 μg/ml | 1 (100) | 10 (67) |  | 0.377 |  | 3 (50) | 5 (56) |  | 0.80 (0.11–6.35) | 0.832 |
| Treatment Duration ≥ 12 months | 1 (100) | 13 (87) |  |  |  | 5 (83) | 8 (89) |  | Ref |  |
| < 12 months | 0 (0) | 2 (13) |  | 0.599 |  | 1 (17) | 1 (11) |  | 1.60 (0.08–31.8) | 0.759 |
| STFX dose, not receiving 200 mg | 0 (0) | 2 (13) |  |  |  | 0 (0) | 1 (11) |  | Ref |  |
| receiving 200 mg | 1 (100) | 13 (87) |  | 0.599 |  | 6 (100) | 8 (89) |  | NA | 0.301 |
| Clarithromycin resistance after therapy (-) | 1 (100) | 11 (73) |  |  |  | − | − |  | − |  |
| (+) | 0 (0) | 4 (27) |  | 0.440 |  | − | − |  | − | − |
| STFX MIC >1 μg/ml | 0 (0) | 2 (13) |  |  |  | 0 (0) | 6 (67) |  | Ref |  |
| ≤1 μg/ml | 1 (100) | 13 (87) |  | 0.599 |  | 6 (100) | 3 (33) |  | NA | 0.003 |
| Surgery (-) | 0 (0) | 15 (100) |  |  |  | 4 (67) | 9 (100) |  | Ref |  |
| (+) | 1 (100) | 0 (0) |  | 0.006 |  | 2 (33) | 0 (0) |  | NA | 0.042 |
| Data are shown as no. of patients (%) for sputum culture conversion. ^a^Odds ratio was not available in some categories because at least one category was zero, resulting in no stable estimate. AFB, acid-fast bacillus; AMK, amikacin; BMI, body mass index; CI, confidence interval.; EMB, ethambutol; MAC-LD, *Mycobacterium avium* complex lung disease; MIC, minimum inhibitory concentration; NA, not available; NB, nodular/bronchiectatic; OR, odds ratio; aOR, adjusted OR; Ref, reference; RIF, rifampin; STFX, sitafloxacin. | | | | | | | | | | |
